# Randomized controlled trial of a primary care–based screening program to identify older women with prevalent osteoporotic vertebral fractures: Cohort for skeletal health in Bristol and Avon (COSHIBA)

**DOI:** 10.1002/jbmr.1478

**Published:** 2011-11-23

**Authors:** Emma M Clark, Virginia Gould, Leigh Morrison, AE Ades, Paul Dieppe, Jon H Tobias

**Affiliations:** 1 Academic Rheumatology, Musculoskeletal Research Unit, University of Bristol, Avon Orthopaedic Centre, Southmead HospitalBristol, UK; 2 School of Social and Community Medicine, University of BristolBristol, UK; 3 Peninsular College of Medicine and Dentistry, University of PlymouthPlymouth, UK

**Keywords:** RANDOMIZED CONTROLLED TRIAL, SCREENING, VERTEBRAL FRACTURES

## Abstract

Approximately 12% of postmenopausal women have osteoporotic vertebral fractures (VFs); these are associated with excess morbidity and mortality and a high risk of future osteoporotic fractures. Despite this, less than one-third come to clinical attention, partly due to lack of clear clinical triggers for referral for spinal radiographs. The aim of this study was to investigate whether a novel primary care–based screening tool could be used to identify postmenopausal women with osteoporotic VFs and increase appropriate management of osteoporosis. A randomized controlled trial was undertaken in 15 general practices within the Bristol area of the UK. A total of 3200 women aged 65 to 80 years were enrolled, with no exclusion criteria. A simple screening tool was carried out by a nurse in primary care to identify women at high risk of osteoporotic VFs. All identified high-risk women were offered a diagnostic thoracolumbar radiograph. Radiographs were reported using standard National Health Service (NHS) reporting, with results sent back to each participant's general practitioner (GP). Participants in the control arm did not receive the screening tool or radiographs. The main outcome measure was self-reported prescription of medication for osteoporosis at 6 months with a random 5% subsample verified against electronic GP records. Secondary outcome was self-reported incidence of new fractures. Results showed that allocation to screening increased prescription of osteoporosis medications by 124% (odds ratio [OR] for prescription 2.24 at 6 months; 95% confidence interval [CI], 1.16 to 4.33). Allocation to screening also reduced fracture incidence at 12-month follow-up (OR for new fracture 0.60; 95% CI, 0.35–1.03; *p* = 0.063), although this did not reach statistical significance. This study supports the use of a simple screening tool administered in primary care to increase appropriate prescription of medications for osteoporosis in postmenopausal women in the UK. © 2012 American Society for Bone and Mineral Research

## Introduction

Osteoporosis is one of the most common diseases affecting elderly women. One of the most serious consequences of osteoporosis is vertebral fractures (VFs), which are common: data from the European Vertebral Osteoporosis Study (EVOS) suggest that 6% to 21% of postmenopausal women have at least one vertebral deformity, the majority of which will be osteoporotic in origin, with an average prevalence of 12%.^1^ These 12% of women have a reduced quality of life,^2^ functional limitations including respiratory compromise,^3^ a modest excess mortality,^4^ and immediate costs to health care providers of between $2000^5^ and $7300.^6^ Perhaps most important, they are at high risk of further vertebral^7^ and other osteoporotic fractures.^8^ However, if these undiagnosed women were prescribed appropriate osteoporosis medications, then expected further fractures could be reduced by between 20% and 50%.^9^, ^10^

Despite this, less than one-third of osteoporotic VFs come to clinical attention.^11^ Possible explanations include inaccurate reporting of spinal radiographs and failure of appropriate recognition and coding of radiograph reports in primary care. However, the probable major reason for this diagnostic failure is that there is lack of awareness of clinical triggers for referral for diagnostic spinal radiographs in patients with possible VFs. General practitioners (GPs) are generally dissuaded from referring patients for radiographs in the UK, and without accurate clinical indicators for prevalent VFs, the great majority of patients are likely to remain undiagnosed until presenting with late clinical sequelae.

We previously carried out a population-based cross-sectional study to define clinical risk factors for identifying women at high risk of prevalent VFs, to serve as a preselection tool for spinal radiographs.^12^ In that study we examined associations between the presence of VF and risk factors for VF ascertained from “hands-on” assessment by a nurse, in 509 women 65 to 75 years old recruited from general practices in southwest UK. Receiver operating characteristic (ROC) curves suggested that the model for predicting more than one VF may have diagnostic utility, in light of predictive values of 0.88 (0.80–0.97). Using a threshold of four, good separation of osteoporosis risk scores was observed according to the presence or absence of more than one VF. If this threshold had been applied to the original population in order to preselect patients for radiographs, this would have reduced the number of X-ray referrals by approximately 70%, while identifying one-half of those with one VF and nearly all of those with more than one VF. We postulated that these four independent clinical predictors could be used as a simple screening tool for identifying women at high risk of more than one VF in a population-based setting.

The aim of this pragmatic randomized controlled trial (RCT) based on the Cohort for Skeletal Health in Bristol and Avon (COSHIBA), which was specifically recruited for this purpose, was to investigate if this simple screening tool would appropriately increase the prescription of medications for osteoporosis. This primary outcome measure was chosen as a proxy for reduction in fracture incidence: although fracture occurrence was also examined, this was specified as a secondary outcome due to our limited power to detect a reduction in fracture risk as measured directly.

## Patients and Methods

The trial was run from the University of Bristol and recruited participants from multiple general practices within the Bristol area of the UK. Practices that took part in the original pilot were not recruited. Ethics approval was obtained from the National Research Ethics Service (REC reference number 07/Q2005/47), and all study participants provided written consent. The study protocol was registered with ClinicalTrials.gov (NCT00463905). The study was funded by the Arthritis Research UK via a Clinician Scientist Fellowship for the principal investigator (E.C.).

### Recruitment of study participants and baseline assessment

**Fig. 1:**
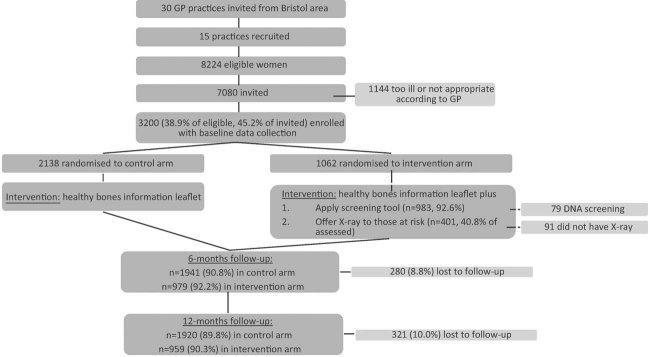
Flow diagram of the trial process based on the CONSORT statement 2010.

Thirty general practices were approached to take part in this study and 15 agreed (Fig. 1). Information was provided to all participating general practices on appropriate management of prevalent VFs. As described previously,^13^ these general practices were from a range of neighborhoods and deprivation scores. Eligibility criteria for individual participants was being female and having a date of birth between January 1, 1927 and December 31, 1942. There were no exclusion criteria, although some primary care physicians did not invite women they thought were unsuitable (such as those with serious illness or cognitive impairment). Baseline data collection was by self-completion of questionnaires prior to randomization. Wording of questions has previously been described.^13^

### Baseline data

Data collected included osteoporosis medications prescribed at point of entry to study, traditional risk factors for osteoporosis such as personal and family history of hip fracture, previous fracture after age 50 years, smoking, early menopause, oral corticosteroids for more than 3 months, and number of falls over the previous 5 years. Highest education achieved qualification was categorized into none/basic matriculation, vocational, O-levels or equivalent, A-levels or equivalent, or university degrees. This scale was similar to that derived for another large population-based cohort in Bristol, the Avon Longitudinal Study of Parents and Children.^14^, ^15^ Data were also collected on current housing tenure. Data on current height and weight were collected by self-completion questionnaires. As previously published,^13^ a random 5% subsample of self-completion questionnaires was validated against electronic general practice records and in general there was good agreement.

### Randomization and masking

After baseline data collection, participants were electronically randomized in a 2:1 ratio in favor of controls. Randomization was stratified by baseline osteoporosis medication prescription, and used block sizes of 6, 9, and 12. Participants' GPs were blinded as to which arm their patient had been assigned. Trial coordination, follow-up data collection, coding, and data entry was done under masked conditions, as was analysis.

### Intervention

All participants received a “healthy bones” leaflet giving information on healthy lifestyles to maintain bone health and reduce falls. Those allocated to the intervention arm were additionally invited to their general practice for a 20-minute appointment with our research nurse trained clinically to the level of a practice nurse. During this appointment, data was collected on the four clinical risk indicators identified in our previous study: height loss, history of previous non-VF, Margolis back pain score, and rib-to-pelvis distance.^12^ If the calculated risk score was below 4 (our predetermined threshold), participants were identified as being at high risk of a prevalent VF, and offered a thoracolumbar radiograph at the nearest hospital. It had been arranged that the radiographs would be reported using standard National Health Service (NHS) reporting techniques and the report would be sent back to the individual participant's GP for further action. This meant that the interaction between the study team and GPs was limited to a meeting at original recruitment of the practice, and provision of information on appropriate management of prevalent VFs. No further communication was made between researchers and GPs: instead usual NHS mechanisms for reporting results were used to mimic any future real-life situation.

### Follow-up assessment

Every 6 months after enrollment, questionnaires were posted to all the participants asking about new prescription of osteoporosis medications and new fractures.

### Outcomes

The primary outcome was self-reported new prescription of medications for treating osteoporosis, assessed at the 6-month follow-up. No false-positive or false-negative self-reported osteoporosis medication prescriptions were identified on the random 5% subsample verified against electronic records. Secondary outcome was self-reported new fractures at 6-month and 12-month follow-up, along with new prescription of medications for treating osteoporosis at the later time point. No single individual could contribute more than one fracture to this secondary outcome.

### Statistical analysis

The planned sample size (*n* = 3860) was based on a power of 80% and a 5% significance level, but actual recruitment was 3200. However, in reality, baseline osteoporosis medication prescribing was 7.3 per 100 women, increasing to 9.9 per 100 women. A post hoc power calculation shows that we still have an 80% power to detect this increase (one-tail test). Statistical analyses were performed (by E.C.) using Stata version 11 (StataCorp, LP, College Station, TX, USA). Intention to treat analyses were used for analysis of the primary and secondary outcomes. Screening effects were presented as differences in percentages of women with new prescriptions for osteoporosis medications or fractures, and compared by study arm using chi-square tests. Odds ratios for new prescription of osteoporosis medications or fractures for those in the screening arm were calculated using logistic regression. The number needed to screen to give one additional prescription was calculated from the usual equation of the reciprocal of the risks of these outcomes in the control minus the screening arm.

## Results

A total of 8224 eligible women were identified. Of these, 1144 were not invited to take part by their GP as it was felt to be inappropriate (medical, psychological, or social reasons) (see Fig. 1). Of the 7080 women invited, 3200 were enrolled (38.9% of eligible or 45.2% of invited) between October 2007 and May 2009, and were assigned to the control arm (*n* = 2138) or screening (*n* = 1062). No data is available on those who declined to take part. Baseline characteristics of participants in the two arms are shown in Table 1. No differences were seen. Median age of participants was 72.1 years (interquartile range [IQR], 69.0–76.1).

**1:** Demographic and Baseline Characteristics of Participants Randomized to Control and Screening

	Control group (*n* = 2138) mean (SD)	Screening arm (*n* = 1062) mean (SD)	*p* Value for difference
Age (years)	72.6 (4.3)	72.7 (4.3)	0.691
Current height (cm) (*n* = 2855)	160.1 (6.6)	160.2 (6.4)	0.582
Current weight (kg) (*n* = 3041)	69.4 (13.5)	69.4 (12.6)	0.999

	Control group *n* (%)	Screening arm *n* (%)	*p* Value for difference
Educational qualifications (*n* = 2896)			0.281
None/basic matriculation	786 (40.5)	400 (41.9)	
Vocational	487 (25.1)	224 (23.5)	
O-levels of equivalent	329 (16.9)	184 (19.3)	
A-levels or equivalent	229 (11.8)	95 (10.0)	
University	111 (5.7)	51 (5.4)	
Housing tenure (*n* = 3012)			0.777
Owned/mortgaged	1737 (86.1)	858 (86.3)	
Private rental	33 (1.6)	18 (1.8)	
Council or HA rental	191 (9.5)	96 (9.7)	
Sheltered, residential, or NH	57 (2.8)	22 (2.2)	
Smoking (*n* = 3154)			0.149
Never	1108 (52.6)	581 (55.4)	
Used to	841 (40.0)	382 (36.4)	
Currently smoking	156 (7.4)	86 (8.2)	
Baseline osteoporosis medication prescription (*n* = 3200)			0.923
Yes	155 (7.3)	78 (7.3)	
No	1983 (92.7)	984 (92.7)	
Age at menopause (*n* = 2942)			0.277
Less than 45 years	558 (28.4)	295 (30.3)	
45 or older	1410 (71.7)	679 (69.7)	
Fractures after age 50 years (*n* = 3199)			0.878
Yes	558 (26.1)	280 (26.4)	
No	1579 (73.9)	782 (73.6)	
Steroids for >3 months (*n* = 3025)			0.426
Yes	162 (8.0)	89 (8.9)	
No	1859 (92.0)	915 (91.1)	
Maternal hip fracture (*n* = 3150)			0.782
Yes	237 (11.3)	117 (11.2)	
No	1664 (79.1)	836 (79.9)	
Do not know	203 (9.6)	93 (8.9)	
Falls (*n* = 3068)			0.155
Never	761 (36.9)	382 (37.9)	
Once every few years	725 (35.2)	371 (36.8)	
Once per year	256 (12.4)	132 (13.1)	
Few times a year	296 (14.4)	111 (11.0)	
Every month or more	23 (1.1)	11 (1.1)	

HA = housing association; NH = nursing home.

**Fig. 2:**
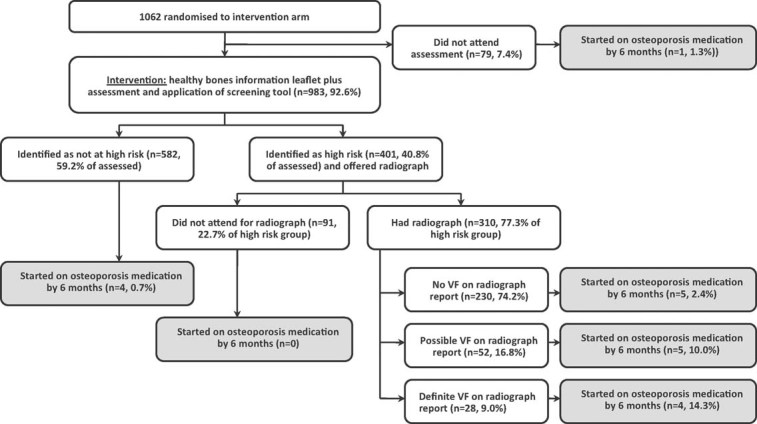
Flow diagram of participants in the intervention arm showing numbers assessed as being at high risk, numbers who had thoracolumbar radiographs, numbers identified with vertebral fractures (VFs), and those started on osteoporosis medication at 6 months.

Trial compliance was generally good, with 92.6% of the screening arm attending the assessment (see Fig. 1). However, of those identified to be at high-risk of VFs (*n* = 401), 22.7% (*n* = 91) did not attend for spinal radiographs (see Fig. 2). The main reason given for nonattendance was the distance needed to travel to the hospital. Of the 310 thoracolumbar radiographs performed 230 (74.2%) were reported as showing no evidence of VFs, 52 (16.8%) were reported as possible VFs (using wording such as “probably a little loss of height,” “possible wedging of a couple of vertebrae,” and “minor depression of the superior endplate”), and 28 (9.0%) were reported as showing definite VFs. For the primary outcome of new prescriptions of osteoporosis medications 92.2% (*n* = 979) of the screening arm and 90 · 8% (*n* = 1942) of the control arm returned the 6-month follow-up questionnaire. The 12-month follow-up was completed by 89.8% (*n* = 1920) and 90.3% (*n* = 959), for the control and screening arms, respectively.

At the 6-month follow-up, allocation to screening increased the odds of prescription of osteoporosis medications by 124% (odds ratio [OR] for prescription 2.24; 95% confidence interval [CI], 1.16–4.33; *p* = 0.016) (see Table 2). Between 6 and 12 months no differences were seen in prescription of osteoporosis medications. There was an association between the presence of VF on the radiograph report (see Table 3 and Fig. 2) and prescription of osteoporosis medication. However, only 26.3% (*n* = 21) of those with definite or possible VFs had been prescribed osteoporosis medication by 6 months: 17.3% of possible and 42.9% of definite. Reporting of definite VF was associated with an increased proportion of new prescriptions for osteoporosis medications at 6 months compared to possible reporting of a VF, or reporting of no VF (*p* = 0.004). Of those who were high risk and had radiographs performed, 64.3% of women prescribed osteoporosis medications within 6 months of starting the study had been identified with definite or possible osteoporotic VF according to the NHS radiograph report. However, some new prescriptions were for women in the intervention arm identified as not at high-risk during the screening, and some prescriptions were for those women for whom no VF was identified on radiograph (see Fig. 2). The number needed to screen to produce one additional prescription for osteoporosis medications within 6 months was 92.

**Table 2:** Osteoporosis Medication Prescription and Fractures in Those in the Control and Screening Arms at 6-Month and 12-Month Follow-Up

	Control arm, *n* (%)	Screening arm, *n* (%)	OR (95% CI)	*p*
Osteoporosis medication prescription				
Within 6 months of joining the study (*n* = 2921)			2.24 (1.16–4.33)	0.016
Yes	17 (0.9)	19 (1.9)		
No	1925 (99.1)	960 (98.1)		
Between 6 and 12 months of joining the study (*n* = 2710)			0.99 (0.45–2.23)	0.998
Yes	18 (1.0)	9 (1.0)		
No	1788 (99.0)	895 (99.0)		
New fractures				
Within 6 months of joining the study (*n* = 2921)			0.87 (0.47–1.61)	0.664
Yes	34 (1.8)	15 (1.5)		
No	1908 (98.2)	964 (98.5)		
Between 6 and 12 months of joining the study (*n* = 2703)			0.28 (0.12–0.67)	0.004
Yes	41 (2.3)	6 (0.7)		
No	1752 (97.7)	904 (99.3)		

CI = confidence interval; OR = odds ratio.

**3:** Prescription of Osteoporosis Medications and Presence of Vertebral Fracture on Thoracolumbar Radiograph Report

	Presence of vertebral fracture on radiograph report (*n* = 310)			
		
	No (*n* = 230)	Possible (*n* = 52)	Yes (*n* = 28)	
On osteoporosis medications at baseline				0.007
Yes	22 (9.6)	4 (7.7)	8 (28.6)	
No	208 (90.4)	48 (92.3)	20 (71.4)	
New prescription of osteoporosis medication by 6 months				0.004
Yes	5 (2.4)	5 (10.0)	4 (14.3)	
No	208 (97.7)	45 (90.0)	24 (85.7)	
New prescription of osteoporosis medication between 6 and 12 months				0.167
Yes	1 (0.5)	1 (2.3)	1 (4.8)	
No	194 (99.5)	43 (97.7)	20 (95.2)	

Values are *n* (%).

At 12 months no difference was seen in new fractures between participants in the control group and those in the screening arm (OR for fracture 0.60; 95% CI, 0.35–1.03; *p* = 0.063). A total of 3.5% of the control arm (*n* = 75) and 2.0% (*n* = 21) of the screening arm fractured between 0 and 12 months. However, between 6 and 12 months of follow-up allocation to screening reduced the odds of occurrence of new fractures by 72% (OR for fracture 0.28; 95% CI, 0.12–0.67; *p* = 0.004). Assessment of site of fracture in control and intervention arms (see Table 4) suggests most of the reduction were forearm fractures.

**4:** Observed Fractures in the Control Arm Compared With Observed and Expected Fractures in the Screening Arm in the First 12 Months

	Control arm (*n* = 2138)	Screening arm (*n* = 1062)	
		
	Observed fractures, *n* (%)	Observed fractures, *n* (%)	Expected fractures, *n*
No fractures	2063 (96.49)	1041 (98.0)	1025
Total fractures	75 (3.51)	21 (1.98)	37
Forearm fractures	29 (1.36)	6 (0.57)	14
Hip fractures	6 (0.28)	3 (0.28)	3
Vertebral fractures	6 (0.28)	1 (0.10)	3
Other fractures	34 (1.59)	11 (1.04)	17

Expected fractures were calculated using the proportions found in the control arm. Fracture data was self-reported by participants.

## Discussion

In this first trial of a screening program for identification of postmenopausal women with VFs we found that, compared to the control group who were not screened, allocation to screening doubled osteoporosis medication prescription and appeared to reduce fractures. The increase in prescriptions were mainly appropriate, since over one-half of new prescriptions in the high-risk group were for those women identified with definite or possible VFs on radiograph. These results suggest that this method of screening for prevalent VFs, which takes a maximum of 20 minutes and can be performed by a practice nurse, should be considered in primary care for all older women.

Our study reports an increase in new osteoporosis medication prescribing over the first 6 months after screening, but not during the second 6 months. This fits with the timing of risk factor assessment and referral for spinal radiographs. It suggests there is a window of opportunity, in which either a patient is appropriately prescribed osteoporosis medications or the opportunity is lost. Although we detected an increase in new prescriptions for osteoporosis medications by 6 months, under 50% of those with definite VF had been treated with osteoporosis medications over this period, despite providing each general practice with written management protocols for prevalent VFs. In order to mimic the real-life situation in which screening was rolled-out by practice nurses, we used a “hands-off” approach with minimal interaction between the research team and GPs. It is well-recognized that changing organizational culture such as that within primary care to improve healthcare performance is challenging,^16^ and the large care gap we report is similar to that seen with other general practice–based interventions for osteoporosis management.^17^ Future implementation of this screening program needs to focus carefully on the response to the radiology report, highlighting this as a key time-point in appropriate management of women with VFs.

The reduction in fractures between 6 and 12 months was unexpected as we were not powered to identify the expected fracture reduction with osteoporosis medication, and this may be a chance finding, although the overall 12-month reduction in fractures approached statistical significance. However, the reduction seen was in forearm and “other” fractures, two categories of fracture whose risk is generally not affected by use of bisphosphonates. Nonetheless, temporally it is plausible that our screening arm contributed to this reduction, perhaps with a combination of increased osteoporosis medication prescribing and other changes such as increased prescribing of calcium and vitamin D which may reduce falls.^18^ An alternative explanation is that allocation to the screening arm changed behavior in the women, such as reducing the amount of activity they performed through fear of fractures due to risk of osteoporosis. However, we feel this is unlikely as a qualitative Interpretative Phenomenological Analysis (IPA) of semistructured interviews with 10 women within the screening arm who were found to be at high risk of VF suggests behavior was not affected.^19^

### Strengths and limitations

To our knowledge, this is the only RCT of a population-based screening program for identification of women with prevalent VFs. In addition, despite the large size of the study population, the tool was simple and quick to perform, and could easily be carried out by a trained nurse. Furthermore, the participants invited to the study were done so on the basis of their age with no exclusion criteria. However, the recruited study cohort only included 38.9% of the eligible women. This likely bias may have implications for generalizability of our results. However, as with most research involving people,^20^ the women who did not take part in our study are likely to be at higher risk for VFs and osteoporosis than the women who did take part. Therefore, if the proportion of women taking part in any future screening could be increased this is likely to mean that the screening will be more effective. Other strengths are the small proportion of participants lost to follow-up, and the analysis of end-points by researchers blinded to screening allocation.

Our follow-up was for a relatively short duration due to practical and financial reasons. Nonetheless, our proxy outcome of prescription of osteoporosis medications suggests that in the longer term this screening tool will reduce fractures and prevent the associated reduction in quality of life. It may also be cost-effective: a preliminary cost-effectiveness analysis suggested a cost per quality-adjusted life year of around £30,000, but further modeling suggests that this would be improved considerably by increasing the proportion of women found to have a VF who are prescribed appropriate medication. More sophisticated modeling is required of subsequent year-on-year benefits of appropriate osteoporosis medication prescription. This will need to incorporate long-term adherence rates, which in the case of bisphosphonates are relatively low,^21^ but may be higher following use of newer parenteral therapies such as zoledronic acid or denosumab.^22^ Ideally, reduction in fractures would be a primary end-point, but this requires large trials of long duration (perhaps 5 to 10 years).

Another limitation is use of self-reported osteoporosis medication prescription and fractures. However, in the 5% random subsample compared against electronic general practice records these data do not suffer any nonrandom misclassification.^13^ Any errors in self-reporting are likely to reduce the strength of any association found toward the null, rather than produce a spurious result. Also, thoracolumbar radiographs impart a dose of radiation equivalent to a year's background radiation, and although one in four women we X-rayed were identified with a possible or definite VF it is important to consider this radiation exposure to the 75% without VF. Alternative methods of imaging for VF are available such as lateral DXA (VFA), but while imparting lower radiation doses they are less easy to interpret than traditional radiographs because of poorer image quality. Finally, although our screening tool had a predictive value of 0.88 (significantly higher than the World Health Organization's Fracture Risk Algorithm [FRAX] score,^23^ at around 0.7), it identifies all women with multiple VFs and approximately one-half of those with one VF, rather than all women with VFs.

### Comparison with other trials

No other trials of screening tools for VFs have been identified. We are aware of no national screening programs in place in any country across the world. However, many areas of the UK have local Fracture Liaison Services (FLS). The general aim of FLS is to help target osteoporosis treatments at patients with the highest absolute risk of fracture in order to maximize the cost-effectiveness of the service.^24^ The key subgroup of people targeted by FLS are those who present with a fracture, usually to secondary care. However, less than one-third of VFs present clinically,^11^, ^25^ so FLS usually miss those with VFs as their first presentation of osteoporosis. Our screening tool could therefore run alongside FLS.

There is increasing interest in developing screening tools for osteoporosis more generally, as shown by the recent emergence of risk assessment tools such as the World Health Organization's FRAX.^23^ FRAX is an online tool developed to allow calculation of an individual's absolute risk of hip or other major osteoporotic fracture over the next 10 years. However, once age and femoral neck bone mineral density (BMD) are known, the additional eight risk factors used in FRAX do not significantly improve the prediction of VFs over the next 4 years.^26^ Our screening tool therefore provides an additional method of identifying those at risk of osteoporosis and future fracture.

### Conclusion and policy implications

In conclusion, compared with use of an information leaflet, our screening tool for VFs increased new osteoporosis medication prescription over the initial 6 months, followed by a decrease in risk of fracture over the subsequent 6 months. However, educational initiatives may be required to improve GPs' understanding of the need to prescribe osteoporosis medication after identification of a VF, if maximal benefit is to be obtained.

## Disclosures

All authors state that they have no conflicts of interest.
